# Optogenetic control of cAMP oscillations reveals frequency-selective transcription factor dynamics in *Dictyostelium*

**DOI:** 10.1242/dev.204403

**Published:** 2025-01-14

**Authors:** Kensuke Yamashita, Kazuya Shimane, Tetsuya Muramoto

**Affiliations:** Department of Biology, Faculty of Science, Toho University, 2-2-1 Miyama, Funabashi, Chiba 274-8510, Japan

**Keywords:** Optogenetics, Gene expression regulation, Signal transduction, Frequency modulation, Biological oscillations

## Abstract

Oscillatory dynamics and their modulation are crucial for cellular decision-making; however, analysing these dynamics remains challenging. Here, we present a tool that combines the light-activated adenylate cyclase mPAC with the cAMP biosensor Pink Flamindo, enabling precise manipulation and real-time monitoring of cAMP oscillation frequencies in *Dictyostelium*. High-frequency modulation of cAMP oscillations induced cell aggregation and multicellular formation, even at low cell densities, such as a few dozen cells. At the population level, chemotactic aggregation is driven by modulated frequency signals. Additionally, modulation of cAMP frequency significantly reduced the amplitude of the shuttling behaviour of the transcription factor GtaC, demonstrating low-pass filter characteristics capable of converting subtle oscillation changes, such as from 6 min to 4 min, into gene expression. These findings enhance our understanding of frequency-selective cellular decoding and its role in cellular signalling and development.

## Teaser

**Summary:** Controlling cAMP oscillation frequency in *Dictyostelium* reveals how signal modulation regulates gene expression and influences cellular decoding mechanisms.

## INTRODUCTION

The temporal dynamics of signalling pathways are crucial for appropriate cellular decision-making across various biological processes. Biological oscillations, such as circadian rhythms and somitogenesis, ensure precise timing through robust signalling networks, even in the face of environmental and genetic perturbations (Isomura and Kageyama, 2014; Pourquie, 2003). In contrast, pulsatile oscillations, such as those in calcium concentrations or kinase activation (e.g. ERK activity), are characterised by spontaneous firing or stochastic initiation triggered by external stimuli (Muta et al., 2018; Smedler and Uhlen, 2014). The features of these oscillatory dynamics – amplitude, frequency, duration, and phase – guide cellular decision-making in proliferation, differentiation, and migration. Modulating these parameters enables cells to adapt to environmental changes, as demonstrated by the frequency modulation of Crz1 in *Saccharomyces cerevisiae* and the regulation of sporulation timing in *Bacillus subtilis* (Cai et al., 2008; Hsu et al., 2019; Levine et al., 2012). These examples emphasise the importance of oscillation parameter modulation in enabling cells to decode environmental signals and make appropriate decisions.

Frequency modulation plays a significant role in the development of the social amoeba *Dictyostelium discoideum*, particularly during its transition from a unicellular to a multicellular stage triggered by starvation. This process is driven by synchronised oscillations of cAMP, which shift from random pulses to coordinated oscillations as aggregation progresses (Gregor et al., 2010; Siegert and Weijer, 1989; Tomchik and Devreotes, 1981). The oscillation frequency increases from approximately 5.6 min during early aggregation to 2.5 min in the loose mound stage, enhancing aggregation and regulating long-range signalling through positive feedback between cellular excitability and cAMP oscillations (Ford et al., 2023; Hashimura et al., 2019). These oscillatory dynamics regulate key transcription factors, such as GtaC and the Hbx5-MybG complex, which shuttle between the nucleus and cytoplasm in response to cAMP oscillations and Erk2 phosphorylation (Cai et al., 2014; Hadwiger et al., 2022; Hao et al., 2024). A 2-min cAMP cycle suppresses GtaC translocation, potentially functioning as a ‘low-pass filter’ that activates GtaC during a 6-min cycle (Cai et al., 2014). This is further supported by the observed suppression of *csaA* transcription during high-frequency cAMP cycles (Corrigan and Chubb, 2014). However, this hypothesis has yet to be directly validated through experimental evidence. Conventional tools, such as periodic cAMP microinjection and caged cAMP, provide precise temporal control (Rietdorf et al., 1998; Westendorf et al., 2013), but they lack the capability to sustain long-term oscillations or achieve the spatial precision required for continuous cAMP oscillations.

Optogenetics is a valuable tool for modulating complex signalling dynamics, including light-induced modifications in signalling pathways and the regulation of gene expression (Arekatla et al., 2023; Izquierdo et al., 2018; Meyer et al., 2023; Toettcher et al., 2013). Photoactivated adenylyl cyclase (PAC) is particularly advantageous, as it enables precise spatial and temporal control of cAMP production. In *Dictyostelium*, PAC has been employed as a powerful tool to analyse precisely the mechanisms by which dynamic spatiotemporal patterns regulate complex processes, such as multicellular development (Chen et al., 2014, 2017; Ford et al., 2023; Tanwar et al., 2017; Westbrook et al., 2023). However, while PAC facilitates cAMP production, real-time tracking of frequency modulations during optogenetic stimulation remains challenging. Flamindo2 and FRET-based sensors enable enhanced real-time observation of cAMP dynamics in live cells (Gregor et al., 2010; Hashimura et al., 2019; Singer et al., 2019), but their requirement for blue-light excitation overlaps with the activation wavelength of PAC, complicating combined experiments.

This study aimed to clarify the functional role of frequency modulation in the development of *Dictyostelium* and the timing of gene expression. We utilised the light-responsive cAMP-synthesising enzyme mPAC from cyanobacteria (Chen et al., 2014) alongside the cAMP biosensor Pink Flamindo (P-Fla) (Harada et al., 2017) to manipulate and observe high-frequency cAMP dynamics. Our approach highlights the potential of optogenetics as a precise tool for modulating signalling pathways, offering valuable insights into the mechanisms of frequency-selective decoding that govern complex cellular behaviours. These findings deepen our understanding of cellular signalling in developmental processes and provide a framework for investigating various biological phenomena.

## RESULTS AND DISCUSSION

### Optogenetic manipulation and monitoring of intracellular cAMP

**Fig. 1.**
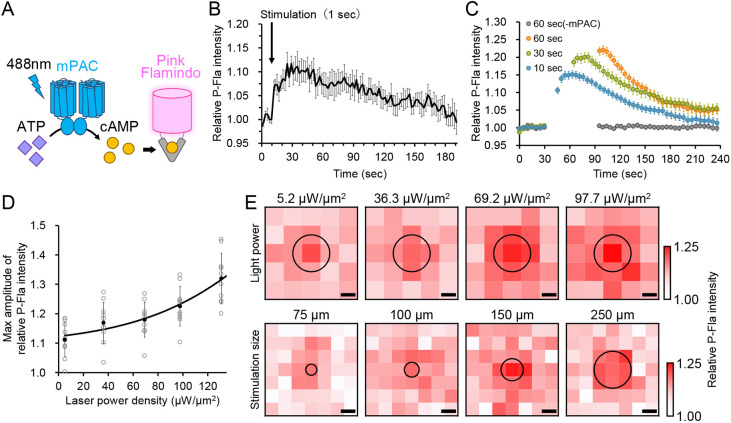
**Optogenetic manipulation and visualisation of intracellular cAMP levels.** (A) Schematic illustrating cAMP production by light stimulation of mPAC and detection using the cAMP sensor Pink Flamindo (P-Fla). (B) Changes in P-Fla fluorescence intensity following a 1-s light stimulation with 69.2 µW/µm^2^ (arrow). Mean fluorescence intensity, normalised to pre-stimulation levels, is plotted against time (*n*=7 cells). Error bars show s.e.m. (C) cAMP level changes in response to varying stimulation durations. Light stimulation commenced at 30 s for the durations indicted (*n*=40-42 cells). −mPAC, negative control. (D) P-Fla intensity in relation to laser power during light stimulation. Error bars represent s.d. (E) Relative changes in intracellular cAMP levels following light stimulation at different light intensities and stimulation area size. The central circle marks the stimulated area, with consistent cell density across all conditions. Scale bars: 100 µm.

To manipulate and monitor intracellular cAMP levels in living cells, we genetically modified cells to express both mPAC and the red-shifted intracellular cAMP indicator P-Fla (Fig. 1A) (Chen et al., 2014; Harada et al., 2017). Although Flamindo2 (Fla2), a yellow-shifted cAMP biosensor, is commonly used in *Dictyostelium* (Ford et al., 2023; Hashimura et al., 2019), we selected P-Fla to avoid spectral overlap with the excitation wavelength of mPAC. By applying extracellular cAMP stimulation and simultaneously monitoring the relative intensities of Flamindo2 and P-Fla, we confirmed that P-Fla reliably detected both cAMP oscillations during cell aggregation and concentration-dependent responses (Fig. S1). Therefore, P-Fla was chosen as the intracellular cAMP sensor for further analyses.

A brief, 1-s optogenetic stimulation in cells co-expressing mPAC and P-Fla triggered a rapid 1.1-fold increase in fluorescence intensity, peaking with a time constant of τ=8.03 s and returning to baseline after 180 s (Fig. 1B). In *Escherichia coli*, a similar stimulation with 0.5 s of light exposure produced a time constant of τ=14 s (Raffelberg et al., 2013), indicating comparable functionality. Extending the stimulation duration elevated intracellular cAMP levels; however, the increases were not proportional to the duration of exposure, with sustained high cAMP levels observed during prolonged stimulation (Fig. 1C). Higher light intensity and larger stimulation area also correlated with increased cAMP levels (Fig. 1D,E), demonstrating that optogenetically induced cAMP dynamics can be visualised under a microscope.

To determine whether optogenetically elevated cAMP could be secreted and induce chemotactic responses via adenylyl cyclase (ACA) activation in neighbouring cells, we mixed ‘inducer cells’ expressing mPAC with ‘sensor cells’ expressing P-Fla without mPAC. Upon optogenetic activation of inducer cells, sensor cells showed increased cAMP levels, leading to chemotactic aggregation (Fig. S2). P-Fla fluorescence revealed a marked increase in the stimulated area compared to controls, with sensor cells peaking at 45 s, compared to 10-15 s in co-expressing cells (Fig. 1B, Fig. S2). This delay likely reflects the time required for extracellular cAMP to activate neighbouring cells. Additionally, increased cell density in the stimulated area supports the conclusion that mPAC-generated cAMP can trigger chemotactic signalling in nearby cells.

Unlike bPAC from *Beggiatoa* sp., mPAC shows higher basal activity under both light and dark conditions (Raffelberg et al., 2013; Stierl et al., 2011), raising concerns regarding cAMP production during non-stimulated periods. Consistent with previous findings, our experiments showed a slight increase in cAMP levels in mPAC-expressing cells, even in the absence of light. However, light-induced cAMP production was greater with mPAC, demonstrating a 36.7-fold increase compared to 28.0-fold with bPAC in oocytes (Raffelberg et al., 2013). These characteristics make mPAC advantageous for precise control of cAMP oscillations, which led to its selection for this study. While P-Fla was effective for monitoring cAMP, it was less sensitive than Fla2, which has higher quantum yields. Another biosensor, R-FlincA, offered high fluorescence intensity (Ohta et al., 2018) but failed to achieve a satisfactory signal-to-noise ratio during optogenetic stimulation, likely due to its high cAMP binding affinity.

### Manipulating the frequency of cAMP oscillations and multicellular development

**Fig. 2.**
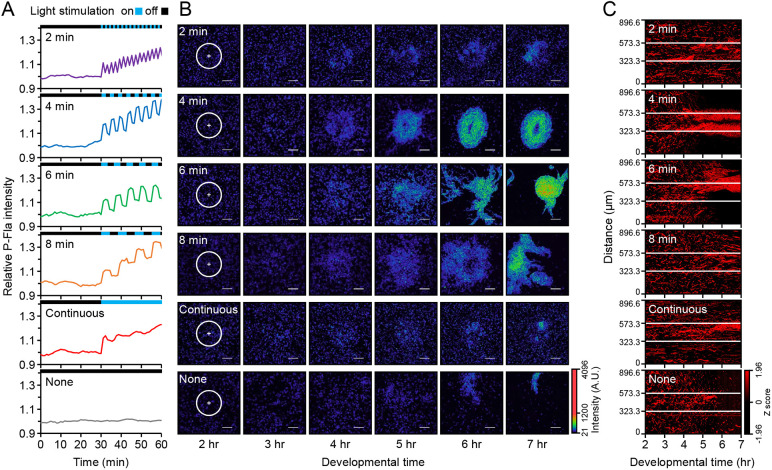
**Manipulation of cAMP oscillation in the formation of aggregation centres.** (A) cAMP oscillation was manipulated by light stimulation during development. Imaging was performed for 30 min without stimulation, followed by periodic stimulation using a 50% duty cycle with 69.2 µW/µm^2^ irradiance. Fluorescence intensity was normalised to the average before stimulation. (B) Aggregate formation efficiency was influenced by the frequency of cAMP oscillation. The rainbow pseudocolour illustrates P-Fla fluorescence intensity (A.U., arbitrary units). Light stimulation was applied to a diameter of 250 µm area as indicated by the white circles. Scale bars: 100 μm. (C) Kymograph of normalised P-Fla fluorescence intensity profiles along the diagonal of the images in B. The region within the white lines delineates the area of light stimulation.

Precise periodic stimulation using optogenetics is crucial for modulating high-frequency cAMP oscillations. We applied continuous or periodic optogenetic stimulation at 2, 4, 6, and 8 min, revealing dynamic cAMP fluctuations corresponding to the stimulation timing (Fig. 2A). Even the fastest 2-min cycles produced consistent cAMP oscillations, although they were shorter than those observed during typical aggregation stages in wild type (Hashimura et al., 2019). This suggests a rapid cAMP synthesis and degradation mechanism. Across all conditions, baseline fluorescence gradually increased, likely due to enhanced fluorescence from three-dimensional cell aggregation and the accumulation of intracellular cAMP.

We analysed whether optogenetically induced cAMP oscillations could mimic aggregation centre formation. Cells with rapid oscillations attract surrounding cells to form aggregation centres, whereas slower oscillating mutants fail to aggregate, leading to cell dispersal (Dormann et al., 2001b). Using a 4-min optogenetic cycle, we observed stable aggregation and rotational cell movements, similar to spontaneous aggregation (Fig. 2B, Movie 1). In contrast, 6- and 8-min cycles formed distinct streams and aggregates that subsequently dispersed outside the stimulated area. Higher frequencies, such as 2-min cycles or continuous stimulation, resulted in smaller cell clumps. Kymographs were used to assess aggregation within the stimulated area (Fig. 2C). This analysis revealed that 4-min oscillations promoted coordinated cell movements within the stimulated area, whereas the 6-min and 8-min cycles led to aggregates extending into the surrounding regions. These results indicate that precise control of cAMP oscillation frequency via optogenetics mimics multicellular development in *Dictyostelium*, highlighting the importance of optimising oscillation frequency for specific cellular responses.

**Fig. 3.**
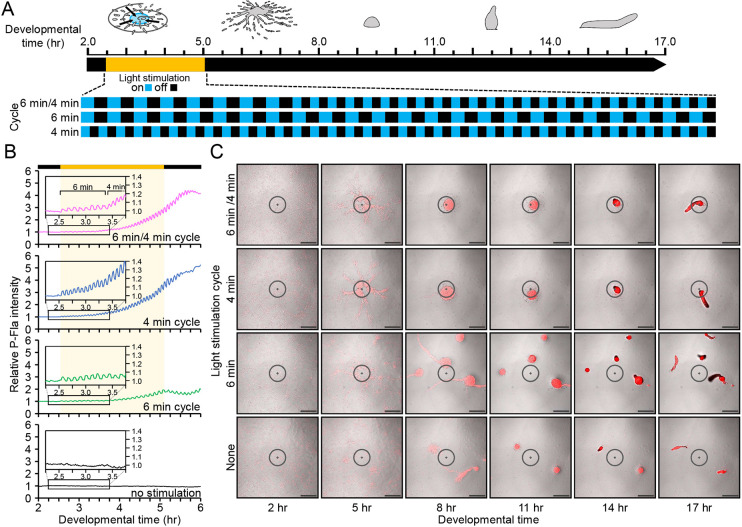
**Frequency coding of multicellular structures in specific regions.** (A) Cells underwent modulated or sustained light stimulations from 2.5 to 5.0 h after starvation, and multicellular structure formation was observed. (B) Changes in relative fluorescence intensity in the stimulated area are shown. Yellow shading indicates the duration of the light stimulation. The inset graph highlights intensity before and after stimulation. (C) Formation of multicellular structures under various stimulation cycles. Diameter of the stimulation area: 250 µm. Scale bars: 250 µm.

We used optogenetic manipulations to investigate whether the formation of multicellular structures depends on frequency modulation rather than fixed oscillation cycles. Light stimulation, either modulated or consistent, was applied during a developmental period of 2.5-5 h, and subsequent developmental processes were observed (Fig. 3A). Intracellular cAMP levels responded reliably to both types of stimulation, with autonomous oscillations continuing post-stimulation (Fig. 3B). When the frequency was modulated from 6 min to 4 min, a single aggregate formed within the stimulation area and underwent typical morphogenesis, progressing from mound to culminant stages (Fig. 3C, Movie 2). Conversely, a consistent 4-min cycle produced similar morphogenesis, whereas a 6-min cycle caused the aggregate to disperse and form multiple small migratory multicellular structures (slugs) outside the stimulated area. To evaluate further the impact of frequency modulation on aggregation, we reduced cell density to 1/10 and applied light stimulation. Modulating the frequency from 6 min to 4 min led to the formation of small multicellular structures (Fig. S3, Movie 3). A consistent 4-min cycle showed limited aggregation, whereas a 6-min cycle increased cell accumulation, which collapsed during multicellular development. Established modulation protocols restored aggregation-defective phenotypes in *acaA* and *cdk8* knockouts (KOs). Both KOs exhibited cell aggregation and cAMP oscillation (Fig. S4). These findings suggest that low-frequency cycles enhance cell accumulation, whereas higher-frequency modulation supports the maintenance of a compact aggregate within the stimulated area.

This optogenetic system was applied across various developmental stages to evaluate its broader applicability. In the growth phase, light stimulation successfully induced cAMP oscillations without initiating cell aggregation (Fig. S5), consistent with the distinct cellular behaviours of this stage compared to other developmental phases. During the slug stage, exposure to blue light caused slugs to halt migration and stand upright, suggesting sensitivity to high-intensity light.

Our optogenetic tool for frequency modulation offers advantages over established methods, such as periodic cAMP microinjection, micropipette assays, and microfluidics (Artemenko et al., 2011; Fujimori et al., 2019; Nakajima et al., 2014; Rietdorf et al., 1998; Westendorf et al., 2013). While these techniques provide cAMP control, they often struggle to sustain long-term and spatially targeted cAMP oscillations. Our tool, however, enables sustained cAMP modulation with precise spatial control, which is particularly valuable for observing three-dimensional developmental processes on agar plates. By applying the appropriate frequency signal to targeted areas, we were able to induce cell aggregation even in regions containing fewer than ten cells. This aligns with findings that the organising centres for aggregation emerge from collective cell behaviour rather than from predetermined founder cells (Gregor et al., 2010; Ohta et al., 2018; Sawai et al., 2005; Vidal-Henriquez and Gholami, 2019). This flexibility in optogenetic manipulation is expected to enhance our understanding of parameter modulation during development. Despite these advantages, our tool cannot fully replicate endogenous ACA functionality. Although mPAC induces cAMP oscillations, the oscillation amplitude remains inadequate in the absence of endogenous ACA, possibly due to a lack of a plasma membrane localisation signal. Endogenous ACA predominantly localises in the posterior region of aggregating cells, specifically in areas associated with the plasma membrane and intracellular vesicles (Kriebel et al., 2003). Future improvements could involve developing a photosensitive version of ACA by incorporating an AsLOV2 domain (Lungu et al., 2012). This modification could enhance oscillation amplitude and fidelity, potentially producing cAMP oscillations that more closely mimic natural patterns.

### Frequency modulation of cAMP oscillations drives GtaC shuttling with low-pass filter characteristics

**Fig. 4.**
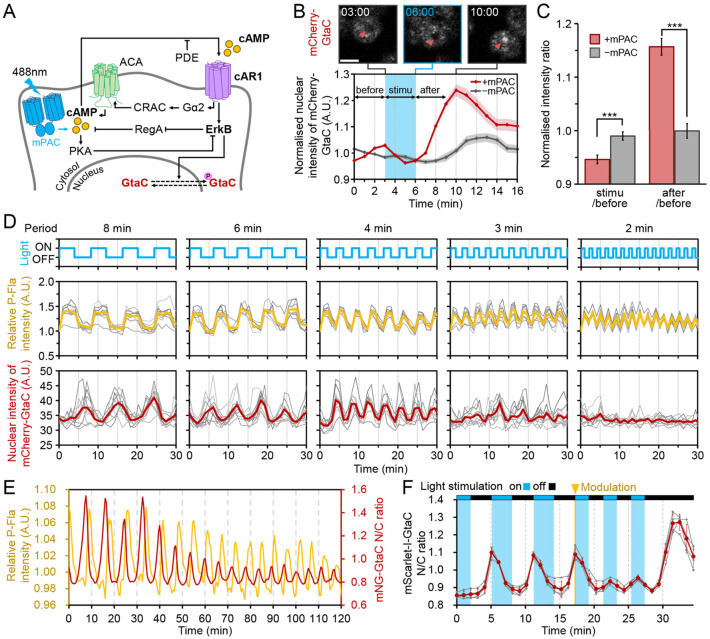
**Low-pass filter characteristics of endogenous GtaC depend on the frequency of cAMP oscillations.** (A) Schematic of the signalling pathway involved in GtaC shuttling. ACA, adenylyl cyclase; cAR1, cAMP receptor 1; CRAC, cytosolic regulator of adenylate cyclase; Gα2, G-protein alpha 2; PDE, phosphodiesterase; PKA, protein kinase A; RegA, intracellular cAMP phosphodiesterase. (B) Response of mCherry-GtaC knock-in cells co-expressing mPAC to 3-min light stimulation. Red arrowheads indicate nuclear positions. Time is in min:sec. Scale bar: 20 µm. In the graph, solid lines represent nuclear fluorescence intensity changes in mCherry-GtaC KI/mPAC (red, *n*=101) and control (grey, *n*=75). Blue shading represents light stimulation, and shaded colours corresponding to each line indicate s.e.m. (C) Comparison of changes in endogenous GtaC localisation with and without mPAC during 3-min intervals before, during, and after light stimulation. Error bars show s.e.m. ***P<0.001 (unpaired *t*-test). (D) Temporal changes in cAMP oscillations and endogenous GtaC in response to periodic stimulation. The upper graph shows the timing of light stimulations. The middle graph shows intracellular cAMP normalised to the initial intensity value. Data represent population averages (yellow and red) and single cells (grey) (*n*=10). (E) Dynamics of cAMP oscillations (yellow) and GtaC shuttling (red) during the spontaneous aggregation process at 7 h of development (*n*=5 cells average). (F) Responsiveness of GtaC to the addition of periodic modulation of cAMP modulation (indicated by a yellow arrowhead). The red line shows the average of the four experiments, with individual traces in grey. A.U., arbitrary units.

Periodic cAMP oscillations regulate the nuclear-cytoplasmic shuttling of GtaC, promoting nuclear export during high cAMP levels and re-entry following dephosphorylation as cAMP decreases (Fig. 4A) (Cai et al., 2014). Using established optogenetic tools, we manipulated GtaC localisation in knock-in cells expressing a red fluorescent protein (Fig. S6). These cells exhibited dynamic GtaC shuttling during aggregation, comparable to, but more consistent than, the GtaC-GFP dynamics reported previously using overexpressed GtaC-GFP (Cai et al., 2014) (Fig. S7, Movie 4). Optogenetic manipulation induced the nuclear export of GtaC, with nuclear re-entry peaking at 4 min post-stimulus (Fig. 4B). Nuclear fluorescence analysis before and after stimulation revealed distinct differences between cells with and without mPAC (Fig. 4C), demonstrating that optogenetic techniques effectively control GtaC localisation at the single-cell level.

We then examined the cAMP oscillation frequencies capable of inducing GtaC shuttling. In the absence of periodic stimulation or under continuous stimulation, GtaC showed no periodic response (Fig. S8). While light stimulation at intervals of 2-8 min produced intracellular cAMP oscillations, GtaC responded only to cycles of 4 min or longer (Fig. 4D). At 2-min intervals, GtaC showed no changes in localisation, while 3-min intervals produced weak amplitude changes in nuclear localisation approximately once every two cycles. These results indicate that GtaC functions as a low-pass filter, acting as a frequency decoder that selectively responds to cycles of 4 min or longer to regulate gene expression timing.

As cAMP oscillations naturally transition to higher frequencies during normal development, we investigated the effects of frequency modulation rather than constant repetitive stimulation. To enable precise analysis, nuclei were labelled with the far-red nuclear marker miRFP670-H2B, which facilitated accurate quantification of GtaC nuclear localisation dynamics. During normal development, GtaC exhibited a stepwise decrease in the amplitude of nuclear localisation as cAMP frequency increased (Fig. 4E). To reconstruct the frequency modulation using the optogenetic tool, the oscillation period was adjusted from 6 min to 4 min. This shift significantly decreased the amplitude of GtaC nuclear localisation while maintaining precise periodicity synchronised with optogenetic stimulation (Fig. 4F).

In *Dictyostelium*, transcription factors serve as developmental timers, responding to cAMP oscillations to regulate gene expression with precision. The concept of the developmental timer was first introduced with GbfA, a transcription factor that gradually accumulates in the nucleus in response to elevated cAMP during development, promoting late-stage gene expression (Firtel, 1995). STATa, another transcription factor, responds to cAMP signalling; however, its dynamics are too slow to match the rapid pace of cAMP pulses (Dormann et al., 2001a). In contrast, the nucleocytoplasmic shuttling of GtaC directly corresponds to cAMP oscillations (Cai et al., 2014), which transition from approximately 5.6 min in early aggregation to 2.5 min in the loose mound stage (Hashimura et al., 2019). Notably, our results demonstrate that GtaC fails to shuttle accurately at oscillation cycles shorter than 4 min, indicating a minimum frequency threshold for an accurate response. This frequency range appears to be crucial for precise gene regulation, such as that of *csaA*, within a narrow developmental timeframe. The reconstruction of frequency modulation provided a more sustained and controlled approach to studying GtaC behaviour, directly validating the ‘low-pass filter’ hypothesis. Unlike earlier studies that relied on cAMP addition and depletion, this setup captured the gradual decline in GtaC nuclear localisation amplitude as cAMP frequency increased. These findings suggest that GtaC operates through an analogue regulatory mechanism, acting as a frequency decoder to fine-tune gene expression in response to developmental signals.

Additional transcription factors, Hbx5 and MybG, have recently been identified in *Dictyostelium*, exhibiting dynamic nucleocytoplasmic shuttling similar to GtaC (Hao et al., 2024). Optogenetic manipulation offers a promising approach for investigating the roles of Hbx5 in response to cAMP oscillation frequency during development. Coordinating multiple transcription factors to decode oscillatory cAMP dynamics and modulate target gene expression in a time-specific manner represents an intriguing avenue for further research.

In conclusion, the application of frequency modulation provides new insights into the developmental timer theory, demonstrating how a 4-min cAMP oscillation period effectively regulates chemotactic aggregation and GtaC shuttling amplitude through the cAMP signalling pathway. This mechanism optimises cellular decision-making in processes such as gene expression, cell aggregation, and multicellular development. These findings enhance our understanding of cellular signalling dynamics and hold significant potential for developing methods to manipulate oscillatory dynamics across diverse cellular contexts.

## MATERIALS AND METHODS

### Cell lines and growth conditions

*Dictyostelium* AX3 strains were grown at 22°C in HL5 axenic medium supplemented with streptomycin, or on SM agar plates with *Klebsiella pneumoniae* (KpGe) lawns. After thawing frozen cell stocks, cells were maintained in the log-phase to prevent transition to the stationary phase and were not cultured for longer than 2 weeks, to minimise variability in growth conditions.

### Generation of expression vectors

For optogenetic activation of cAMP signalling, mPAC was expressed using either the extrachromosomal vector act15::mPAC-YFP (Chen et al., 2014) or the newly constructed vector pTM2559, where the original promoter of pDM358 (Veltman et al., 2009) was replaced with coaA-P obtained from pDM1208 (Paschke et al., 2018). The cAMP sensor, Pink Flamindo (Harada et al., 2017), was integrated into the extrachromosomal vectors pDM304 and pDM358, resulting in the creation of pTM1272 and pTM1273, respectively. The HK12neo_Flamindo2 construct was used as a yellow fluorescent cAMP biosensor, referred to as Flamindo2 (Hashimura et al., 2019). To evaluate GtaC shuttling, nuclear markers were visualised using codon-optimised mNeonGreen (mNG) (Paschke et al., 2018) or miRFP670 (Shcherbakova et al., 2016) fused to histone H2Bv3. Both P-Fla and miRFP670 were synthesised by a gene synthesis service (IDT and Twist Bioscience), with codon-optimised for expression in *Dictyostelium* (Table S1). The expression vectors used in this study are listed in Table S2.

### Generation of KO and knock-in mutants using CRISPR/Cas9 techniques

Knock-in or KO cell lines were generated by designing gRNAs specifically targeting the *cdk8*, *gtaC*, and *cinD* loci. The Cas-designer standalone version of the CRISPR RGEN tool was used following the methodology described in previous studies (Ogasawara et al., 2022; Yamashita et al., 2021). Chemically synthesised gRNAs were prepared as paired oligonucleotides with AGCA or AAAC overhangs (Table S3), and subsequently inserted into either the all-in-one CRISPR/Cas9 vector (pTM1285) (Sekine et al., 2018) or the SpRY all-in-one vector (pTM1668) (Asano et al., 2021) using the Golden Gate Assembly system. The resulting CRISPR/Cas9 vectors were designated as pTM1701, pTM1901, and pTM1933 (Table S4). Donor DNAs used for knock-in mutants was amplified by PCR with primers and templates outlined in Tables S5 and S6. Each donor DNA fragment contained homologous sequences of 60 bp or longer. CRISPR vectors, either alone or in combination with donor DNA, were introduced into AX3 cells via electroporation. For the GtaC imaging, cells were tagged with a near-infrared fluorescent protein, miRFP670, to label nuclei. The donor DNA for this purpose comprised a 2.0 kb fragment containing the *act15* promoter and *act8* terminator flanking the miRFP670-H2B sequence. In total, 14 new cell lines incorporating knock-ins or KOs were established, following a previously described methodology (Table S7).

### Cell preparation for live imaging and optogenetic manipulation

For standard optogenetic experiments, cells were plated on agar at a cell density of 2.6×10^5^ cells/cm^2^, and the supernatant KK2 phosphate buffer (16.5 mM KH_2_PO_4_, 3.9 mM K_2_HPO_4_) was carefully removed after 10 min of incubation. The cells were then incubated in the dark at 22°C until they reached the 2-h developmental stage. Unless otherwise specified, cells at this 2-h stage were used for optogenetic manipulation experiments. For imaging the sensor and inducer cells, equal amounts of each were mixed at a density of 1.6×10^5^ cells/cm^2^. For vegetative cells prepared for optogenetic light stimulation, cells were plated in HL5 medium at a density of 2.0×10^5^ cells/cm^2^ in glass-bottom chambers and incubated in the dark for 1 h prior to imaging and stimulation. For optogenetic manipulation of GtaC shuttling, mCherry-GtaC/[act15]:mPAC cells were plated at a density of 1.0×10^5^ cells/cm^2^, and mScarlet-I-GtaC/[coaA]:mPAC cells at 2.6×10^5^ cells/cm^2^, and then incubated for up to 3 and 4 h, respectively. To visualise miRFP670-H2B, agar was supplemented with 50 µg/ml biliverdin (30891, Sigma-Aldrich). Approximately 30 min prior to imaging, agar was cut into approximately 1-cm squares. The cell-adhering surface was then placed face down in glass-bottomed dishes (Delta T Culture Dishes, Bioptechs).

Before inducing development, cells in the exponential growth phase were washed with KK2. Cells lacking *cdk8* were suspended in development buffer (5 mM Na_2_HPO_4_, 5 mM KH_2_PO_4_, 2 mM MgCl_2_, 1 mM CaCl_2_) and pulsed with 60-80 nM cAMP every 6 min for 5 h. For observing multicellular development, cells were plated on 1.5% agar (Difco Bacto-agar) in KK2 at densities of 5.2-10.4×10^5^ cells/cm^2^ in plastic dishes. For later developmental stages, the agar was placed with the cell-side up in the dish. Mineral oil (M8410, Sigma-Aldrich) was applied to the agar surface to prevent desiccation.

### Imaging and optogenetic conditions

Time-lapse images of live cells were captured using an inverted microscope (Eclipse Ti, Nikon) equipped with a confocal laser system (Nikon A1R, Nikon). During optogenetic manipulation, fluorescent images were captured using a galvanometric scanner. For other confocal imaging applications, a piezo stage combined with resonant scanning was utilised to enable rapid *z*-direction imaging. Both dry and oil immersion objectives were employed, including the Plan Apo λ 10×/0.45 NA, Plan Apo λ 20×/0.75 NA, Plan Fluor 40×/1.30 NA, and Plan Apo λ 60×/1.40 NA (Nikon). Fluorescent tags or probes were visualised using the following excitation wavelengths: Flamindo2 and mNeonGreen with a 488-nm solid-state laser; mCherry, mScarlet-I, and P-Fla with a 561-nm laser; and miRFP670 with a 640-nm laser. The photoactivation feature of the confocal laser system was used to activate mPAC. To calculate the power density of blue light (488 nm) stimulation, the laser power, measured with a silicon photodiode sensor (PD300-3W-V1, Ophir), was divided by the focal spot size calculated based on the Rayleigh criterion. Unless stated otherwise, the standard light stimulation intensity was 36.3 µW/µm^2^. The fluorescence intensity of P-Fla was calibrated within the microscopy system by adding serial dilutions of cAMP or activating mPAC with 6 s of light stimulation. Changes in intracellular cAMP levels under varying light intensities and stimulation area sizes were analysed by measuring fluorescence intensity after 1 min of light stimulation. For experiments involving cAMP frequency modulation and multicellular development, a 30-min pre-stimulus imaging was conducted for normalising analysis data. Periodic stimulation was applied using a 50% duty cycle, in which the light was on for half of each cycle and off for the remaining half, to maintain high levels of intracellular cAMP. For continuous stimulation, the laser intensity was reduced to 5.2 µW/µm^2^ to minimise cytotoxic effects from prolonged exposure. Time-lapse images were captured at 1-min intervals during light stimulation. A circular region with a diameter of 250 µm at the centre of the image field was designated as the stimulation area. In contrast, for mCherry-GtaC KI/mPAC and mScarlet-I-GtaC KI/mPAC cells, the entire imaging field, represented by a square box, served as the stimulation region.

### Image analysis

Imaging data were analysed using NIS-Elements software (AR Analysis version 5.01.00, Nikon) and Volocity software (version 6.3, PerkinElmer). NIS-Elements was primarily used to quantify the mean fluorescence intensity of P-Fla within the stimulated area, with measurements normalised to baseline data collected 30 min prior to light stimulation. Volocity software was used to quantify the fluorescence intensity and cellular density, specifically in the context of P-Fla and GtaC shuttling. Nuclear markers enabled automated cell tracking analysis, allowing quantification of the nuclear-to-cytoplasmic (N/C) ratio in individual cells. A dose-response curve for calibrating P-Fla fluorescence intensity was generated using four-parameter logistic curve fitting with the drc package in R. Time-series data in Fig. 4E, including P-Fla fluorescence intensity and the GtaC N/C ratio, were smoothed using a Savitzky–Golay filter from the ‘signal’ package in R, with a 5-frame window size, to enhance signal clarity and minimise noise.

## Supplementary Material

10.1242/develop.204403_sup1 Supplementary information
