# Supplementary material for: Optogenetic control of cAMP oscillations reveals frequency-selective transcription factor dynamics in Dictyostelium
Source: Development. 2025 Jan 14;152(1):dev204403. doi: 10.1242/dev.204403 (PMC11829771; doi:10.1242/dev.204403)
Supplement: Supplementary information [file develop-152-204403-s1.pdf]

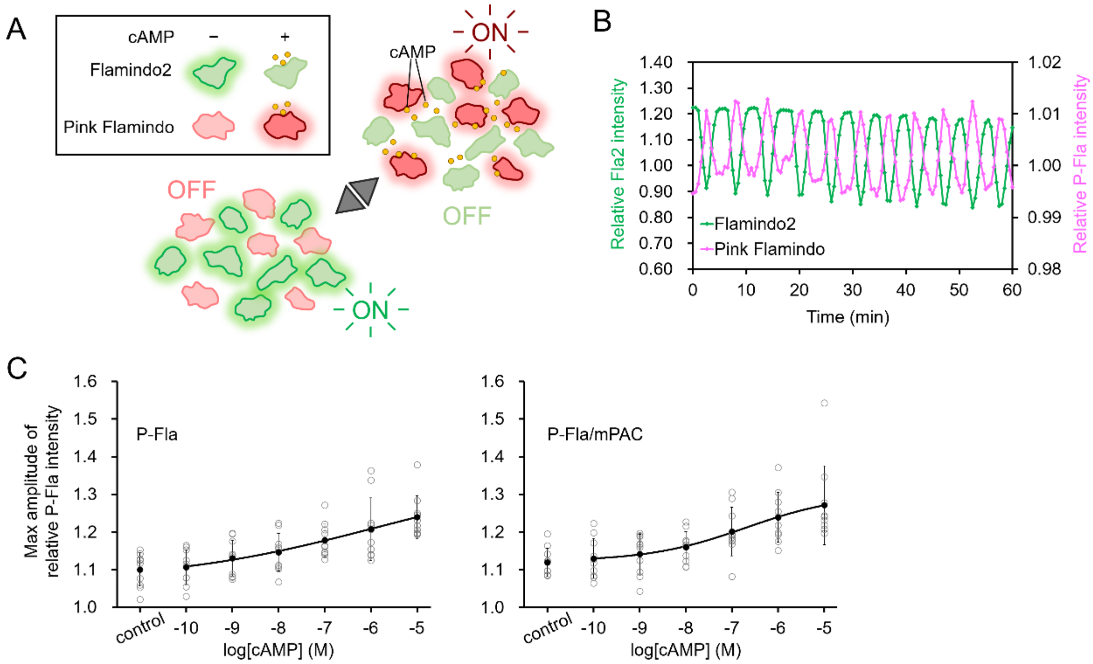

Fig. S1. A Comparison of two cAMP sensors, Flamindo2 and Pink Flamindo.

A) Flamindo2 (Fla2) functions as a turn-off fluorescence sensor, decreasing in brightness upon binding intracellular cAMP, whereas Pink Flamindo (P-Fla) acts as a turn-on sensor, increasing in brightness when interacting with cAMP. **B)** Quantification of intracellular cAMP oscillations during the aggregation stage using a combination of cells expressing Fla2 and P-Fla. **C)** Maximum P-Fla fluorescence intensity against extracellular cAMP concentration.

Development • Supplementary information

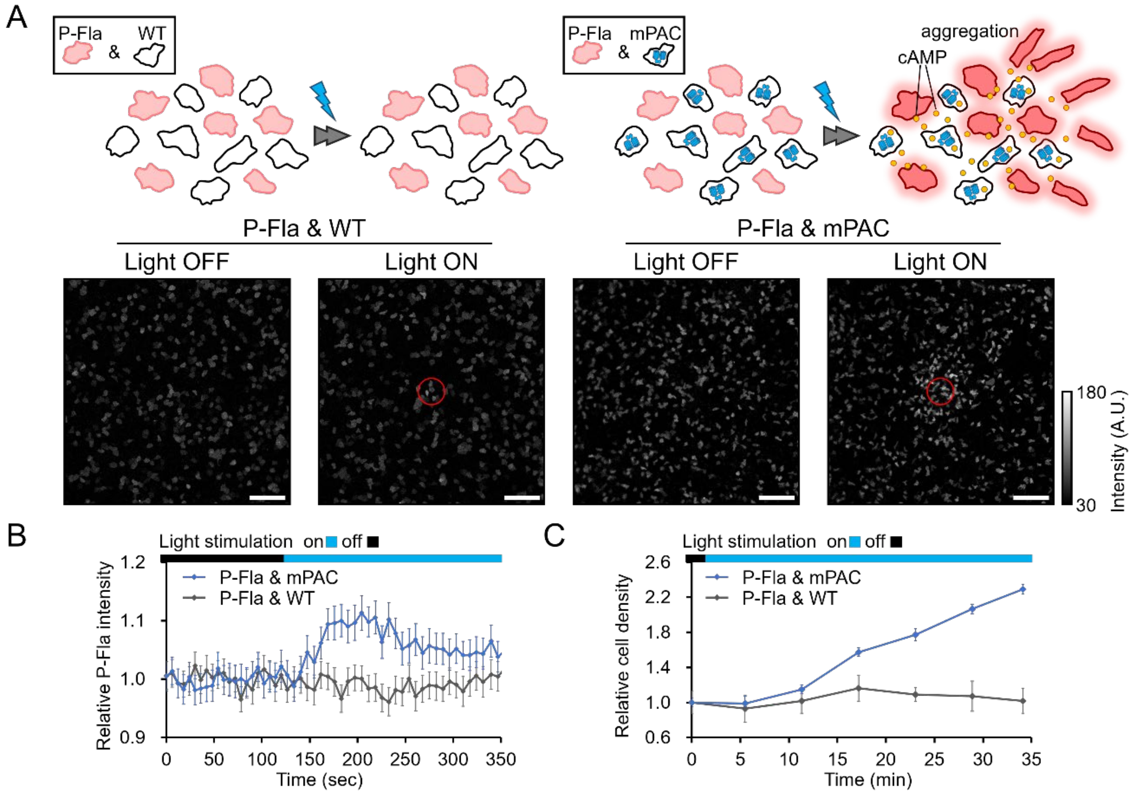

Fig. S2. Changes in intracellular cAMP levels triggered by neighbouring cells expressing mPAC.

A) Verification of the physiological function of cAMP derived from mPAC. Sensor cells expressing P-Fla were mixed in equal proportions with either control wild-type (WT) cells or inducer cells expressing mPAC, both plated at a density of 1.6 × 10^5 cells/cm^2. Only sensor cells are visible in greyscale. Light stimulation was applied in a 75 µm diameter region, delineated by the red circle. Scale bars: 100 µm. **B)** The fluorescence intensity of individual P-Fla cells in the stimulated area was quantified. Data at each time point were normalised by dividing it by the average fluorescence intensity measured before continuous light stimulation. Bars: SE. n = 57–74 cells per data point. **C)** Temporal changes in cell density within the stimulated area. Bars: SE. n = 3.

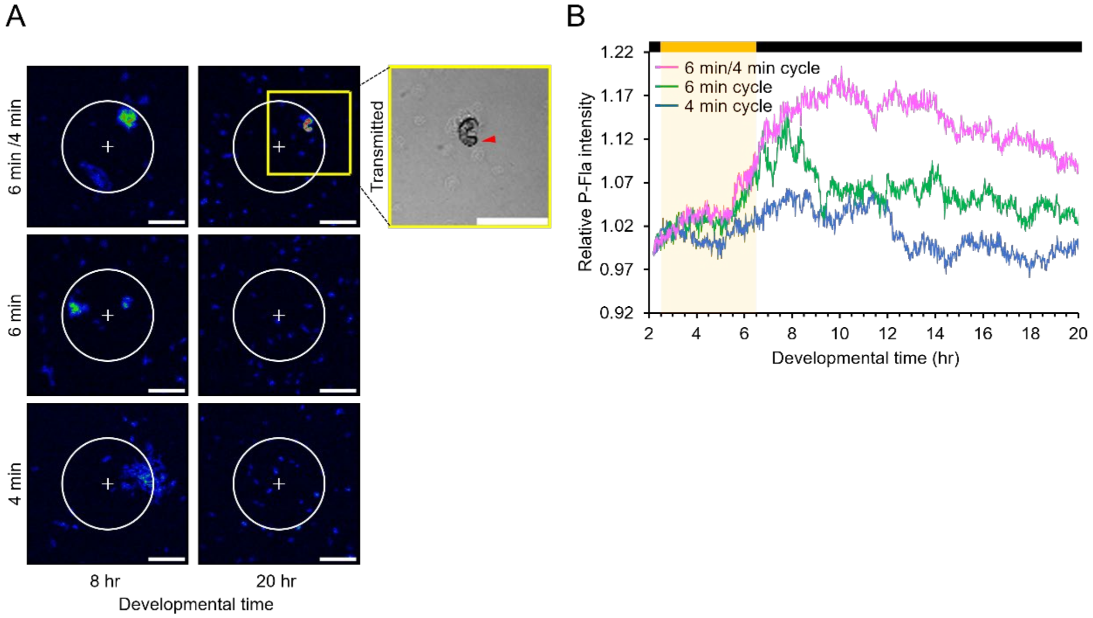

Fig. S3. Frequency-dependent formation of multicellular structures under low cell density conditions.

A) Frequency modulation of cAMP oscillations through light stimulation induced the formation of small multicellular structures in low-density cell populations. Snapshots from each stimulus cycle display P-Fla fluorescence intensity at 8 and 20 hours of development, represented in rainbow pseudocolour. A transmitted-light image provides an enlarged view of the multicellular structure formed within the stimulated area when the cycle was modulated from 6-minute to 4-minute. Light stimulation was applied to a 250 μm diameter area, outlined by white circles. Scale bars: 100 μm. The red arrowhead indicates the tip of the multicellular structure. Cells were prepared at a density of 2.6×10^4 cells/cm^2, which represents 1/10 the standard density typically used in these experiments. **B)** P-Fla fluorescence intensity within the light stimulation area was normalised using data from a 20-minute pre-stimulus imaging period. The yellow shading represents the duration of light stimulation. Fluorescence intensity was low in the 4- and 6-minute cycles (blue and green), due to aggregate collapse. However, frequency modulation from 6 minutes to 4 minutes induced multicellular formation in the stimulated area, maintaining high fluorescence intensity (magenta).

Development • Supplementary information

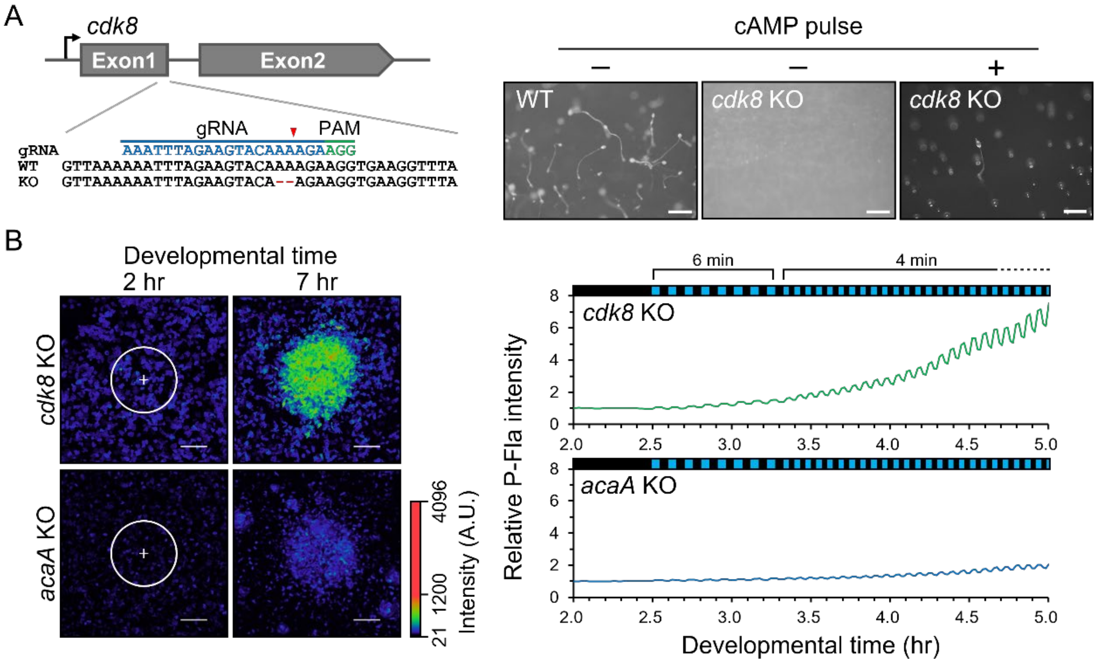

Fig. S4. Restoration of aggregation defects through light manipulation of cAMP oscillations.

A) Establishment of *cdk8* knockout (KO) cell lines and their phenotypic characteristics. A gRNA for CRISPR/Cas9 was designed to target the first exon, indicated in blue. The protospacer adjacent motif (PAM) sequence is highlighted in green, and the mutation insertion site is marked in red. The phenotype of *cdk8* KO showed aggregation defects, which were rescued upon treatment with extracellular cAMP pulses. Scale bars: 1 mm. **B)** Light manipulation with periodic modulation from 6 to 4 minutes was applied to *cdk8* and *acaA* KO strains exhibiting aggregation defects. Responses to light manipulation were examined in cells untreated with extracellular cAMP pulses. The stimulated area is indicated by a white circle, and the graph illustrates the changes in cAMP levels within the stimulation area. Scale bars: 100 μm.

Development • Supplementary information

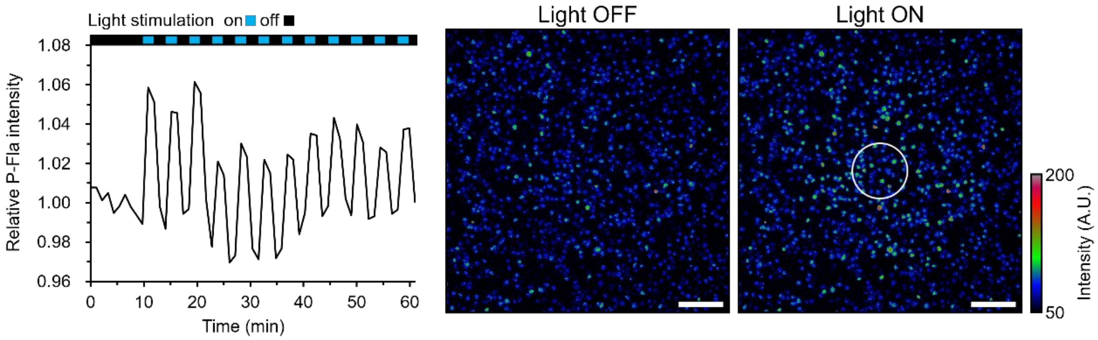

Fig. S5. Periodic manipulation of cAMP levels during the vegetative stage. Light stimulation was applied to P-Fla/mPAC cells at 4-minute cycles during the vegetative stage. P-Fla fluorescence intensity was normalised to data collected 10 minutes prior to light stimulation. Snapshots were captured immediately before and after light stimulation. The white circle represents the light stimulation area, with a diameter of 124.1 μm. Scale bars: 100 μm.

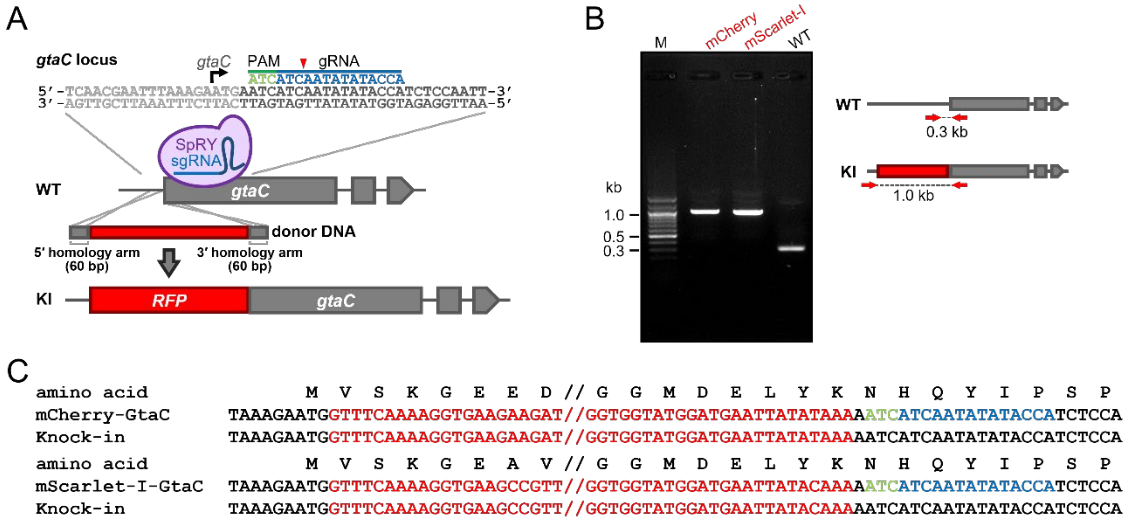

Fig. S6. Knock-in of a gene encoding red fluorescent protein at the *gtaC* locus.

A) Schematic representation of the knock-in strategy for integrating the red fluorescent protein gene at the *gtaC* locus using the CRISPR/Cas9 system. **B)** PCR validation of the knock-in strain was performed using primers targeting regions upstream and downstream of the red fluorescent protein insertion site. The knock-in strain yielded a PCR product with an increased length corresponding to the size of the inserted fluorescent protein. **C)** Nucleotide sequencing of the *gtaC* locus in the knock-in mutants confirmed the integration, with the sequence encoding the red fluorescent protein is highlighted in red.

Development • Supplementary information

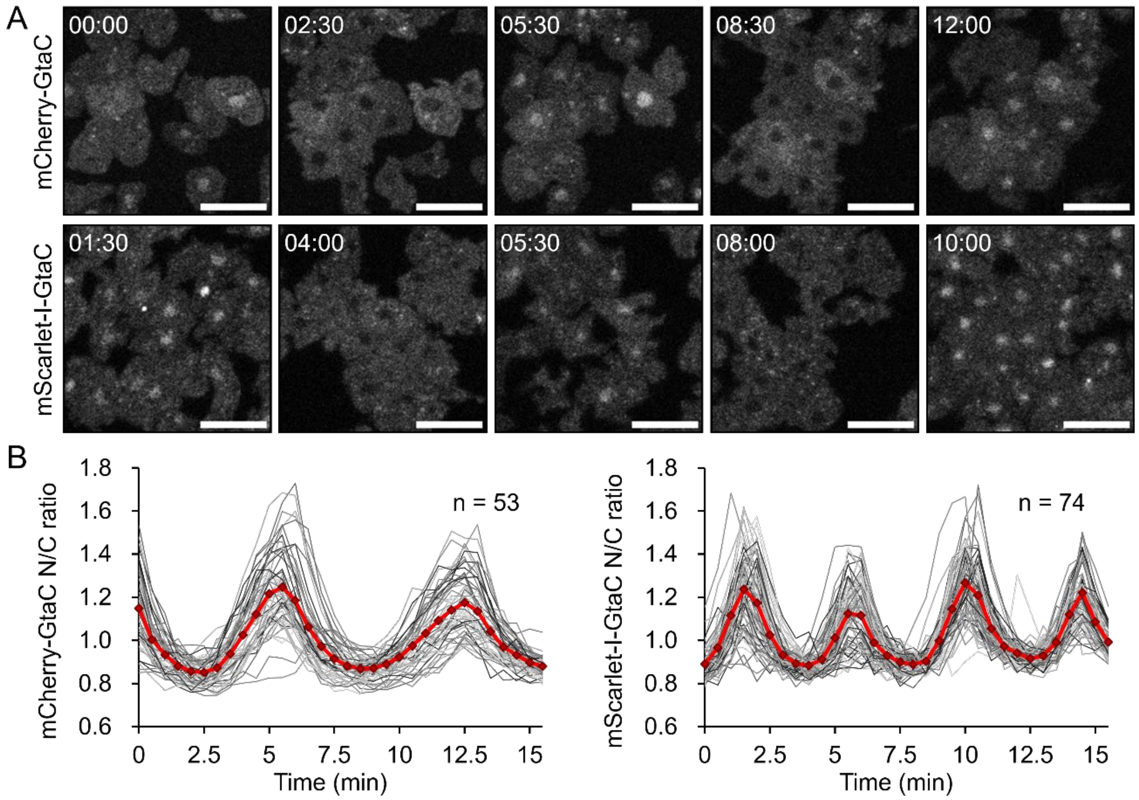

Fig. S7. Visualisation of endogenous GtaC with a red fluorescent tag.

A) Nucleocytoplasmic shuttling of GtaC was monitored using red fluorescent protein. Time is in min:sec. Scale bars: 20 μm. **B)** Population average (red) and individual cell traces (grey) are shown for mCherry-GtaC (n = 53 cells) and mScarlet-I-GtaC (n = 74 cells).

Development • Supplementary information

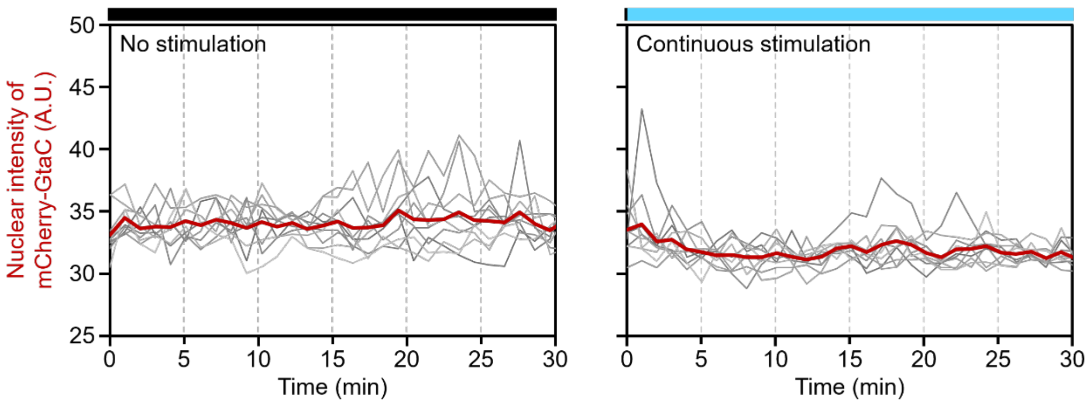

Fig. S8. Localisation kinetics of mCherry-GtaC in response to no stimulation and continuous stimulation.

Light manipulation was applied to cell populations at 2 hours of development for a duration of 30 minutes. Graphs display the mean nuclear fluorescence intensity (red) and single-cell traces (grey) of mCherry-GtaC (n = 10 cells). The bars at the top of the graph represent with stimulation (blue) and without stimulation (black).

Table S1. Codon-optimised nucleotide sequences for Pink Flamindo and miRFP670.

Description	Sequence (5'-3')
**Pink Flamindo**	**ATG**GTGAGCAAAGGTGAAGAAAACAATATGGCAATTATTAAAGAATTTATG<br>CGTTTTAAAGTTCATATGGAAGGTAGCGTTAATGGCCATGAATTTGAAATT<br>GAAGGTGAAGGTGAAGGAAGACCATATGAAGCATTTCAAACTGCTAAACT<br>TAAAGTTACTAAAGGAGGACCTCTACCTTTTGCATGGGATATTCTTAGTCC<br>ACAATTTATGTATGGTAGTAAAGTTTATATTAAACATCCAGCTGATATTCCA<br>GATTATTTCAAACTTAGTTTTCCAGAAGGATTTCGTTGGGAAAGAGTTATG<br>AATTTTGAAGATGGAGGTATTATTCATGTTAATCAAGATAGTAGTCTTCAAG<br>ATGGAGTGTTTATCTATAAAGTTAAACTAAGAGGAACAAATTTTCCTAGTG<br>ATGGACCAGTTATGCAAAAAAAAACAATGGGATGGGAACAATGGAGAGAT<br>CCACCACTTCTTAGTCAAAGAGGACCTGATGCACTTCTTACAGTTGCACT<br>TAGAAAACCTCCAGGACAAAGAACTGATGAAGAACTTGATCTAATTTTTG<br>AAGAACTTCTTCATATTAAAGCAGTTGCACATCTTAGTAATAGTGTTAAAAG<br>AGAACTAGCAGCTGTACTACTTTTTGAACCACATAGTAAAGCAGGTACTG<br>TACTATTTAGTCAAGGTGATAAAGGTACCAGTTGGTATATTATCTGGAAAG<br>GTAGTGTTAATGTTGTAACACATGGTAAAGGACTTGTTACAACTCTACATG<br>AAGGAGATGATTTTGGACAACTTGCGCTTGTTAATGATGCACCAAGAGCT<br>GCTACAATTATTCTAAGAGAAAATAACTGTCATTTTCTTAGAGTTGATAAAC<br>AAGATTTTAATAGAATCATTAAAGATGTTGAATTTAAAGTAGAAACTGTAGA<br>TGAAAGAATGTATCCTGAAGATGGAGCACTTAAAAGTGAAATTAAGAAAA<br>GACTTAAACTTAAAGATGGAGGTCATTATGCAGCTGAAGTTAAAACAACTT<br>ATAAAGCTAAAAAACCTGTTCAACTTCCAGGAGCATATATTGTAGATATTAA<br>ACTAGATATTGTTAGTCATAATGAAGATTATACAATTGTAGAACAATATGAAA<br>GAGCTGAAGGTAGACATAGTACAGGTGGTATGGATGAACTATATAAA**TAA**
**miRFP670**	**ATG**GTAGCAGGTCACGCCAGTGGATCTCCAGCCTTCGGAACCGCCTCT<br>CACAGTAATTGTGAGCACGAGGAAATACACTTGGCCGGAAGTATTCAACC<br>ACATGGTGCTTTATTGGTTGTTTCTGAACATGATCACCGTGTTATCCAAGC<br>CTCAGCTAATGCTGCTGAATTTCTTAATTTAGGTAGTGTTTTAGGTGTTCC<br>ATTAGCCGAAATAGATGGTGATCTTTTAATAAAGATCCTTCCACACCTTGA<br>TCCAACTGCTGAGGGAATGCCTGTTGCAGTTAGATGTAGAATTGGAAATC<br>CATCAACAGAGTATTGTGGTCTTATGCACCGTCCACCAGAGGGTGGTTTA<br>ATTATTGAGTTGGAACGTGCAGGACCAAGTATTGATCTTTCAGGTACTTTG<br>GCTCCTGCTTTAGAAAGAATTAGAACAGCAGGATCATTAAGAGCACTTTG<br>TGATGATACTGTACTTTTGTTTCAACAATGTACTGGATATGATCGTGTAATG<br>GTTTATCGTTTTGATGAGCAAGGTCATGGTTTAGTATTTTCTGAGTGTCAT<br>GTACCAGGTCTTGAGTCATACTTTGGAAATCGTTACCCATCATCAACTGTT<br>CCTCAAATGGCAAGACAACTTTACGTTAGACAAAGAGTTCGTGTTTTAGT<br>AGATGTTACTTATCAACCTGTTCCATTGGAGCCTAGATTATCTCCATTAAC<br>CGGACGTGATTTAGATATGTCAGGTTGTTTCCTTCGTTCTATGAGTCCTTG<br>TCACCTTCAATTTTTGAAAGATATGGGAGTAAGAGCTACATTGGCTGTTAG<br>TCTTGTTGTAGGAGGAAAATTATGGGGTCTTGTTGTTTGTCATCATTATCT<br>TCCAAGATTTATACGTTTCGAGTTACGTGCTATCTGTAAGAGATTAGCCGA<br>ACGTATCGCCACTCGTATAACTGCACTTGAAAGT

Start and stop codons are highlighted in bold.

Table S2. Expression vectors used in this study.

Vector	Description	Backbone	Drug Resistance	Reference
**pDM304**	extrachromosomal expression vector	–	G418	(1)
**pDM326**	extrachromosomal expression vector	–	Blast	(1)
**pDM358**	extrachromosomal expression vector	–	Hyg	(1)
**HK12neo_Flamindo2**	[act15]: Flamindo2	HK12neo	G418	(2)
**act15::mPAC-YFP**	[act15]: mPAC-YFP	pB17S-EYFP	G418	(3)
**pTM1272**	[act15]: Pink Flamindo	pDM304	G418	This study
**pTM1273**	[act15]: Pink Flamindo	pDM358	Hyg	This study
**pDM1208**	[act15]: mCherry	–	G418	(4)
**pTM2035**	[act15]: mNeonGreen	pDM326	Blast	This study
**pTM2036**	[act15]: mNeonGreen-H2Bv3	pTM2035	Blast	This study
**pTM2075**	[act15]: miRFP670	pDM326	Blast	This study
**pTM2115**	[act15]: miRFP670-H2Bv3	pTM2075	Blast	This study
**pTM2554**	extrachromosomal expression vector with coaA promoter	pDM358	Hyg	This study
**pTM2559**	[coaA]: mPAC	pTM2554	Hyg	This study

Blast: Blasticidin S, Hyg: Hygromycin B

Table S3. Oligonucleotides for gRNAs and sequencing primers.

Primer	Gene	Sequence (5'-3')	Purpose
**P1878**	*cdk8*	agcaAAATTTAGAAGTACAAAAGA	gRNA sense
**P1879**	*cdk8*	aaacTCTTTTGTACTTCTAAATTT	gRNA antisense
**P1880**	*cdk8*	CTTCCATTCTCTCTATGATACT	Screening-Fw
**P1887**	*cdk8*	TATAGATTAGACGTTCAAGAG	Screening-Rv
**P2143**	*gtaC*	agcaTGGAGATGGTATATATTGAT	gRNA sense
**P2144**	*gtaC*	aaacATCAATATATACCATCTCCA	gRNA antisense
**P2151**	*gtaC*	AAAATCAATAACCAACTCTCGCA	Screening-Fw
**P2152**	*gtaC*	TGGTCGCTATAAATTGGAGATGGT	Screening-Rv
**P2263**	*cinD*	agcaTGGACGTTCAAACTAAATGT	gRNA sense
**P2264**	*cinD*	aaacACATTTAGTTTGAACGTCCA	gRNA antisense
**P1186**	*cinD*	GAATCAGTTGTATCTAAAGATGG	Screening-Fw
**P155**	*cinD*	AACAGCTTGACTTGGGATGG	Screening-Rv
**P2282**	*mCherry*	TGGGTTGGGAAGCATCATCA	Sanger sequencing
**P1441**	*mCherry*	AGAGGATCCTTTATATAATTCATCCATAC	Sanger sequencing
**P2168**	*mNeonGreen*	GCAGCAATGGTAGATGGTTCAG	Sanger sequencing
**P2167**	*mNeonGreen*	ACTTCTACACCAATCAGCAGCTG	Sanger sequencing
**P2721**	*miRFP670*	ATTGAGTTGGAACGTGCAGG	Sanger sequencing
**P2722**	*miRFP670*	ACCATGACCTTGCTCATCAA	Sanger sequencing
**P2260**	*H2Bv3*	ACTAGATCTATGGTATTCGTTAAAGGTCA AAAG	Sanger sequencing
**P2140**	*H2Bv3*	ACTACTAGTTTAGTTTTTGCTTTCAGTTGG	Sanger sequencing
**P3008**	*mScarlet-I*	ACTGGATCCATGGTTTCAAAAGGTGAAGC	Sanger sequencing
**P3009**	*mScarlet-I*	ACTAGATCTCTTATATAATTCATCCATACC ACCTGT	Sanger sequencing

Lowercase letters of the gRNA represent overhang sequences for Golden Gate Assembly.

Table S4. CRISPR/Cas9 vectors for generating mutants.

Plasmid	Target gene	Backbone	Cas9 Type	Reference
**pTM1701**	*cdk8*	pTM1285	SpCas9	This study
**pTM1901**	*gtaC*	pTM1668	SpRY	This study
**pTM1933**	*cinD*	pTM1668	SpRY	This study

Table S5. Primers for generating donor DNAs.

Primer	Description	Sequence (5'-3')
**P2149**	mNeonGreen left-arm	aacaaccaatcaaataaaacaacaataaggaggagattgtattctcaacg aatttaaagaATGGTTTCAAAAGGAGAAGAAGATA
**P2150**	mNeonGreen right-arm	tacattgtgtacaccagagttttggtcgctataaattggagatggtatatattga tgattTTTATACAACTCATCCATTCCCAT
**P2413**	mCherry left-arm	aacaaccaatcaaataaaacaacaataaggaggagattgtattctcaacg aatttaaagaATGGTTTCAAAAGGTGAAGAAGATA
**P2414**	mCherry right-arm	tacattgtgtacaccagagttttggtcgctataaattggagatggtatatattga tgattTTTATATAATTCATCCATACCACCTG
**P2415**	mScarlet-I left-arm	aacaaccaatcaaataaaacaacaataaggaggagattgtattctcaacg aatttaaagaATGGTTTCAAAAGGTGAAGC
**P2416**	mScarlet-I right-arm	tacattgtgtacaccagagttttggtcgctataaattggagatggtatatattga tgattTTTGTATAATTCATCCATACCACC
**P2261**	miRFP670-H2Bleft-arm	ttatttagttagtaatagttataaaacaaattgtatattaatttaaaatcaaataa aagaaCATTCTCGAGACTAGAGCTAG
**P2262**	miRFP670-H2B right-arm	ggtggccaattttaaattaatttttttaaaaaaaattaataaattttataatttgat atgttacatttagtttgaacgtccatCTTTTTCGAAATCGATAAGC

Lowercase letters represent homologous sequences of target genes.

Table S6. Plasmid DNAs used as PCR templates.

Plasmid	Description	Reference
**pDM1208**	[act15]: mCherry	(4)
**pTM2035**	[act15]: mNeonGreen	This study
**pTM2115**	[act15]: miRFP670-H2Bv3	This study
**pTM2537**	pUPD2_mScarlet-I_AATG_GCTT	NBRP Nenkin ID: G90679

Development • Supplementary information

Table S7. Cell lines used in this study.

Strain	Description	Parental Strain	Vectors Used	Target Gene	Drug Resistance
**AX3**	*Dictyostelium discoideum* Parental strain	–	–	–	non
**dTM690**	Fla2	Ax3	HK12neo_ Flamindo2	–	G418
***acaA*-**	*acaA* KO NBRP Nenkin ID: S00004	–	–	*acaA*	Blast
**dTM887**	P-Fla	Ax3	pTM1272	–	G418
**dTM922**	P-Fla/mPAC in *acaA* KO	*acaA*-	act15::mPAC-YFP pTM1273	–	G418/Hyg
**dTM942**	mPAC in *acaA* KO	*acaA*-	act15::mPAC-YFP	–	G418
**dTM1078**	P-Fla/mPAC	Ax3	act15::mPAC-YFP pTM1273	–	G418/Hyg
**dTM1106**	mPAC	Ax3	act15::mPAC-YFP	–	G418
**dTM1210**	*cdk8* KO	Ax3	pTM1701	*cdk8*	non
**dTM1212**	P-Fla/mPAC in *cdk8* KO	dTM1210	act15::mPAC-YFP pTM1273	–	G418/Hyg
**dTM1410**	mNeonGreen-GtaC KI	Ax3	pTM1901	*gtaC*	non
**dTM1484**	mCherry-GtaC KI	Ax3	pTM1901	*gtaC*	non
**dTM1538**	mCherry-GtaC KI/mPAC	dTM1484	act15::mPAC-YFP	–	G418
**dTM1590**	mCherry-GtaC KI/mNG-H2B	dTM1484	pTM2036	–	Blast
**dTM1715**	miRFP670-H2B	dTM1410	pTM1933	*cinD*	non
**dTM1814**	mNG-GtaC KI/ P-Fla	dTM1715	pTM1273	–	Hyg
**dTM2015**	miRFP670-H2B	Ax3	pTM1933	*cinD*	non
**dTM2135**	mScarlet-I-GtaC KI	dTM2015	pTM1901	*gtaC*	non
**dTM2151**	mScarlet-I-GtaC KI/mPAC	dTM2135	pTM2559	–	Hyg

KI: Knock-in; Blast: Blasticidin S; Hyg: Hygromycin B

Development • Supplementary information

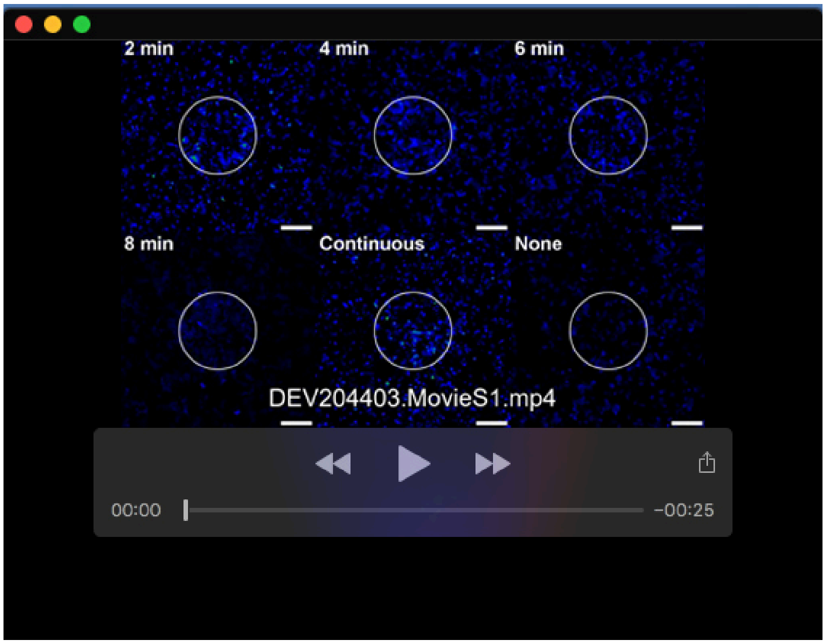

Movie 1. Optogenetic manipulation of aggregate formation.
Designated areas (white circles) were irradiated with blue light at specific frequencies (indicated in the top left of each panel) to examine chemotactic aggregation. Scale bars: 100 μm.

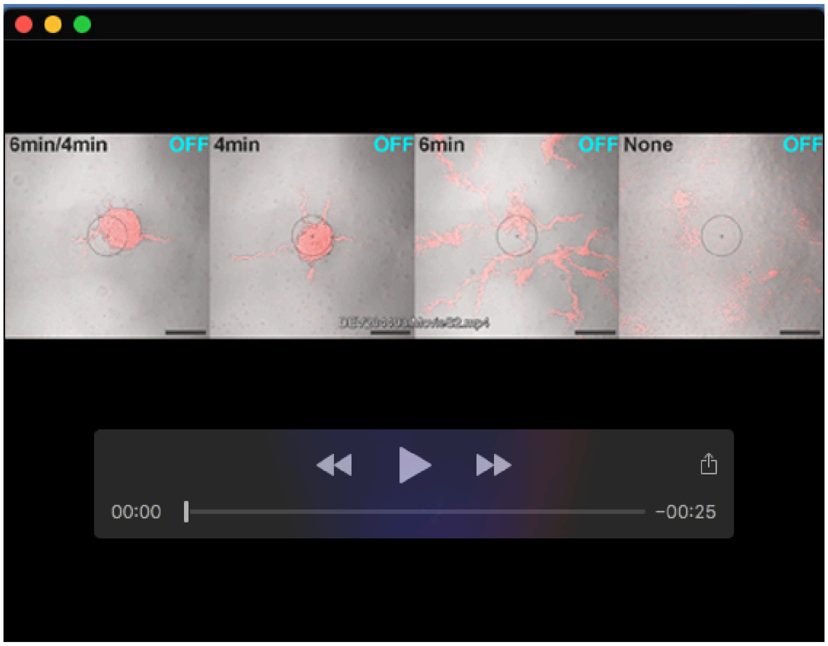

Movie 2. Optogenetic manipulation of multicellular formation in specific regions.
The formation of multicellular structures in the stimulated area is shown under different frequencies of optogenetic manipulation. Both 6-min/4-min and 4-min stimulation cycles initiated multicellular structure formation within the stimulated area. The upper-left corner of each panel displays the stimulation cycle, while the upper-right corner indicates the ON/OFF timing. The diameter of the stimulation area and scale bars are both 250 μm.

Development • Supplementary information

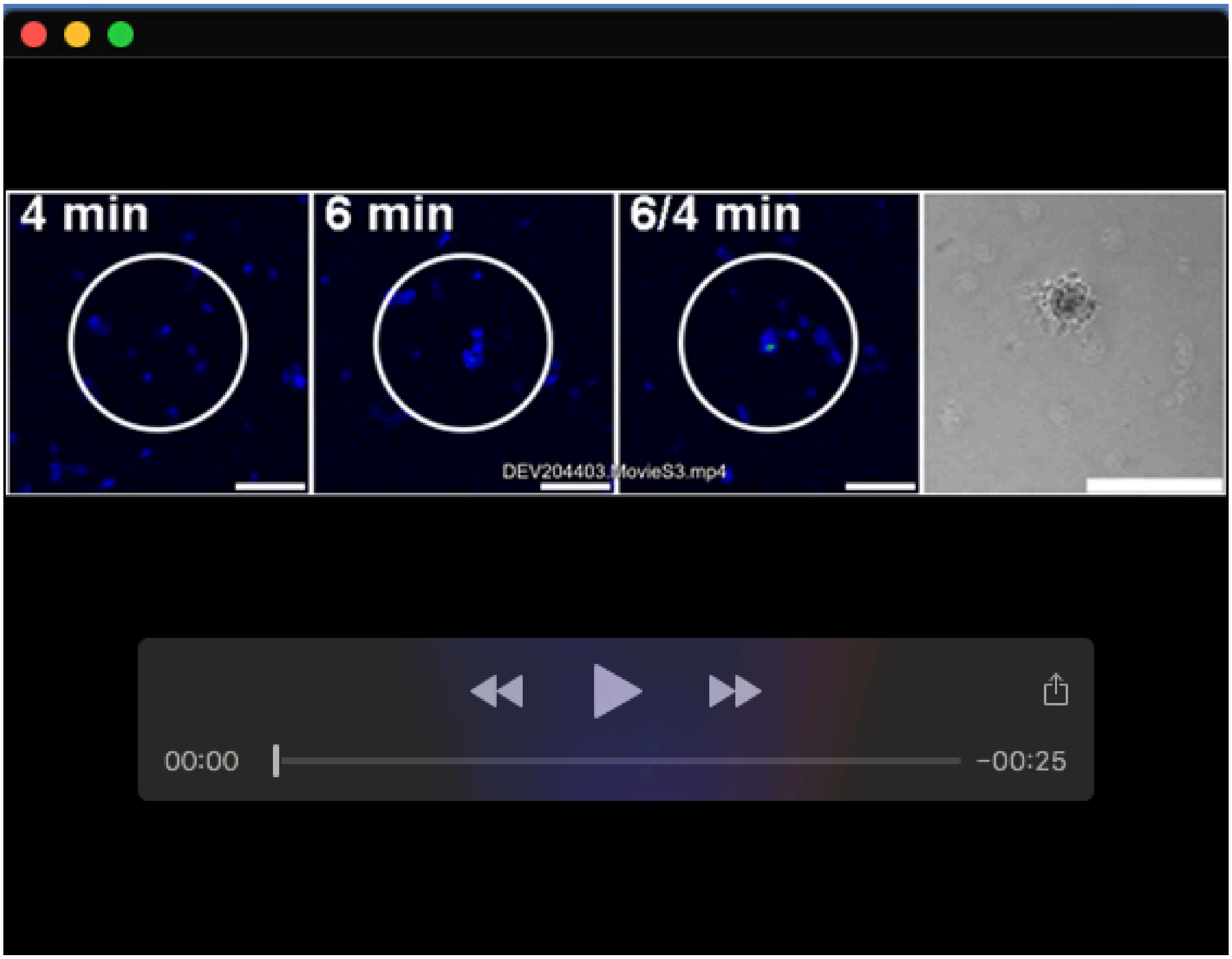

Movie 3. Effectiveness of frequency modulation of cAMP oscillations in low-density cell populations.
Frequency modulation from 6-minute to 4-minute promoted multicellular structure formation in low-density cell populations. White circles indicate the stimulation area with a diameter of 250 µm. A yellow box highlights the area where a small multicellular structure formed, with an enlarged transmitted light image shown in the right panel. Scale bars: 100 µm.

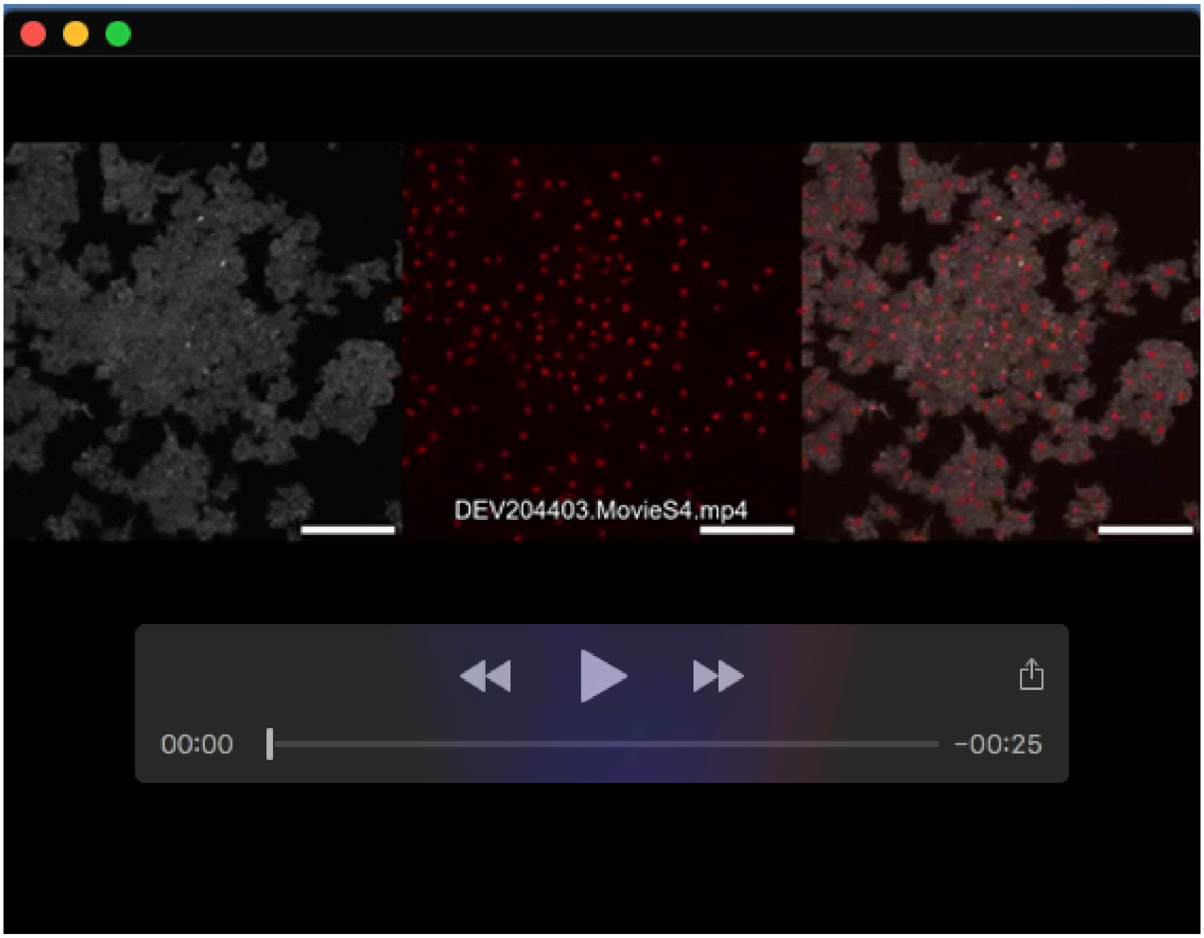

Movie 4. Spontaneous nucleocytoplasmic shuttling of endogenous GtaC. Spontaneous shuttling of mScarlet-I tagged GtaC during cell aggregation process is displayed in the left panel. The middle panel shows H2B labelled with miRFP670 for nucleus identification. Marged images in the right panel link GtaC shuttling with nuclear positions. Scale bars: 50 µm.

SI References

1. Veltman DM, Keizer-Gunnink I, & Haastert PJ (2009) An extrachromosomal, inducible expression system for Dictyostelium discoideum. Plasmid 61(2):119-125.
2. Hashimura H, Morimoto YV, Yasui M, & Ueda M (2019) Collective cell migration of Dictyostelium without cAMP oscillations at multicellular stages. Commun Biol 2:34.
3. Chen ZH, Raffelberg S, Losi A, Schaap P, & Gartner W (2014) A cyanobacterial light activated adenylyl cyclase partially restores development of a Dictyostelium discoideum, adenylyl cyclase a null mutant. J Biotechnol 191:246-249.
4. Paschke P, et al. (2018) Rapid and efficient genetic engineering of both wild type and axenic strains of Dictyostelium discoideum. PLoS One 13(5):e0196809.
